# Addiction to smartphones and social media among young doctors

**DOI:** 10.1192/j.eurpsy.2025.986

**Published:** 2025-08-26

**Authors:** N. Rmadi, A. Hrairi, I. Ben Hnia, R. Affes, O. Walha, F. Dhouib, M. Hajjaji, K. Jmal Hammami

**Affiliations:** 1 Occupational medicine department, Hedi chaker university hospital, University of Sfax; 2 Family medicine department, University of Sfax, Sfax, Tunisia

## Abstract

**Introduction:**

Smartphones and social media have become increasingly common in recent years. However, uncontrolled use can have negative effects on physical and mental health.

**Objectives:**

To evaluate the prevalence of social media and smartphone addiction in a group of young doctors

**Methods:**

Descriptive cross-sectional study of a population of young doctors. Socio-professional and medical data were collected using a self-administered questionnaire on Google Forms. The Bergen Social Media Addiction Scale (BSMAS) was used for screening social media addiction, with a score of ≥24 indicating problematic use. The Smartphone Addiction Scale Short Version (SAS-SV) was adopted to determine the degree of smartphone addiction, where a very high score of >40 suggests severe smartphone addiction.

**Results:**

Our population consisted of 64 young doctors. Among them, 63.8% were female. BSMAS and SAS-SV means were respectively 16.14 ± 5.54 and 28.91 ± 7.62. A social media problematic use was assessed in 13% of cases. We identified severe smartphone addiction in 6.7% of cases. In bivariate analysis, female sex was associated with a higher BSMAS score (p=0.046) and younger age was associated with severe smartphone addiction (p=0.049). Moreover, strong correlation was found between smartphone and social media addiction (p=0.00, r=0.79).

**Conclusions:**

Given the potential negative impacts on mental and physical health, it is crucial for healthcare institutions to implement strategies that promote balanced technology use and raise awareness about the risks associated with addiction. Addressing these issues can ultimately enhance the well-being of medical professionals, fostering a healthier work environment conducive to effective patient care.

**Disclosure of Interest:**

None Declared

